# Nitrofurantoin Susceptibility and the Landscape of Multidrug-Resistant Gram-Negative Uropathogens in Kidney Transplant Recipients During the First Post-Transplant Year

**DOI:** 10.3390/antibiotics15080769

**Published:** 2026-08-10

**Authors:** Aminah Alghamdi, Ehab Hammad, Fatimah S. Alhamlan, Layla Alharbi, Reem Almaghrabi, Fatimah W. Alsmail, Taghreed A. Hafiz

**Affiliations:** 1 Clinical Laboratory Sciences Department, College of Applied Medical Sciences, King Saud University, Riyadh 12372, Saudi Arabia; amalghamdi@ksu.edu.sa; 2 Translational Research in Infectious Diseases, King Faisal Specialist Hospital and Research Center, Riyadh 11211, Saudi Arabia; falhamlan@kfshrc.edu.sa (F.S.A.); lalharbi8@kfshrc.edu.sa (L.A.); 3 Kidney and Pancreas Health Centre, Organ Transplant Center of Excellence, King Faisal Specialist Hospital and Research Center, Riyadh 11211, Saudi Arabia; ehhammad@kfshrc.edu.sa (E.H.); ramaghrabi@kfshrc.edu.sa (R.A.); falsmail@kfshrc.edu.sa (F.W.A.); 4 College of Medicine, AL Faisal University, Riyadh 11211, Saudi Arabia

**Keywords:** urinary tract infection, kidney transplantation, antimicrobial resistance, multidrug resistance, *Escherichia coli*, *Klebsiella pneumoniae*, *Pseudomonas aeruginosa*, antimicrobial stewardship

## Abstract

**Background:** Multidrug-resistant (MDR) Gram-negative urinary tract infections (UTIs) frequently complicate the first post-transplant year in kidney transplant (KTX) recipients. This study characterized patterns of uropathogen susceptibility, MDR prevalence, and predictors of MDR UTI in this population. Methods: In a single-center retrospective cross-sectional study (KFSHRC, Riyadh; October 2024 to December 2025), 83 adult first-transplant recipients with a first-year Gram-negative UTI were included for analysis. Susceptibility was determined using VITEK 2/CLSI criteria, and MDR was defined according to Magiorakos/ECDC criteria. Results: Nitrofurantoin susceptibility varied significantly by species, with 89.2% in *E. coli* compared to 15.0% in *K. pneumoniae* (*p* < 0.001), supporting its use as a carbapenem-sparing option for *E. coli* but not for *K. pneumoniae*. Carbapenems and amikacin retained near-complete activity, whereas cephalosporins, ciprofloxacin, and trimethoprim-sulfamethoxazole exhibited resistance rates of 45 to 68%. *K. pneumoniae* (49.4%) and *E. coli* (44.6%) were the predominant uropathogens, and MDR accounted for 63.9% of episodes (95% CI 53.1 to 73.4). MDR was associated with UTI recurrence, prolonged hospitalization, and reduced microbiological eradication (69.8% vs. 86.7%). UTI recurrence was the only independent predictor of MDR status (adjusted OR, 3.99; 95% CI, 1.46 to 10.89; *p* = 0.007). No UTI-associated mortality occurred within one year. Conclusions: Species-directed therapy, such as nitrofurantoin for *E. coli* and carbapenems or aminoglycosides for broader coverage, combined with local surveillance and antimicrobial stewardship, is essential to preserve treatment options and protect graft function.

## 1. Introduction

Kidney transplantation (KTX) is widely regarded as the optimal treatment for individuals with end-stage renal disease (ESRD), offering improved survival rates and quality of life relative to long-term dialysis. Although advances in surgical techniques and immunosuppressive therapies have been achieved, post-transplant infections continue to pose significant clinical challenges. Among these, urinary tract infections (UTIs) are the most prevalent and substantially contribute to patient morbidity and increased healthcare resource utilization [1]. The incidence of UTIs following KTX varies, with estimates suggesting that 30–60% of recipients experience at least one episode within the first year. Factors contributing to this increased risk include surgical manipulation of the urinary tract, anatomical and functional alterations, and the recipient’s immunosuppressed state. Recurrent and complicated UTIs in this population can lead to serious complications, such as graft dysfunction, acute rejection, and higher mortality rates [2].

The microbiological profile of UTIs in KTX recipients is similar to that of the general population, but KTX recipients have a higher prevalence of opportunistic and antimicrobial-resistant organisms. Bacteria are the primary etiological agents of UTIs in this group, whereas fungal and viral infections are less common [3]. Gram-negative bacteria, primarily *Escherichia coli* and *Klebsiella pneumoniae*, are the most common pathogens, with rising infection rates driven by MDR organisms, including extended-spectrum β-lactamase (ESBL)-producing Enterobacterales and carbapenem-resistant bacteria. These pathogens significantly limit therapeutic options and are frequently associated with poorer clinical outcomes [4]. Global estimates of MDR uropathogen burden in KTX recipients differ significantly by region and methodology. A systematic review and meta-analysis of the Middle East reported a pooled UTI prevalence of 37.9% among renal transplant recipients, with *E. coli* as the predominant isolate (57.5%) [5]. Another review of Iranian cohorts reported a pooled UTI prevalence of 32.6% and identified *E. coli* as the leading Gram-negative pathogen [6]. Data from Saudi Arabia are limited. A recent multicenter retrospective study from Jeddah and Madinah found that 15.3% of screened transplant recipients developed recurrent UTIs, with *K. pneumoniae* as the most frequent isolate (42.9% of episodes) [7]. In contrast, an earlier cross-sectional cohort study from a South Asian transplant center, published in a Saudi-affiliated journal, reported a lower proportion of MDR episodes (27.1% of episodes) than both the present study and the Jeddah/Madinah cohort [8]. This variability among Gulf and Middle Eastern populations highlights that MDR prevalence, pathogen distribution, and susceptibility patterns cannot be generalized across centers or countries. Single-center, contemporaneously interpreted Clinical Laboratory Standard Institute (CLSI) antibiograms remain essential for guiding local empirical therapy.

General Saudi Arabian UTI surveillance data underscore the necessity for transplant-specific analyses. A large cross-sectional study of 1082 urinary isolates from a tertiary hospital in Jazan, southwestern Saudi Arabia, primarily involving non-transplant community and hospital patients, identified *E. coli* as the predominant uropathogen (47.97%), followed by *K. pneumoniae* (24.58%). ESBL-producing isolates accounted for 30.1%, while carbapenemase-producing Enterobacterales comprised only 1.9% of isolates [9]. The pathogen distribution and resistance profiles in this general population differ substantially from those observed in the immunosuppressed kidney transplant cohort, where *K. pneumoniae* was the most common isolate and 63.9% of isolates were MDR. These findings indicate that antibiogram data from general or community UTI populations are not directly applicable to kidney transplant recipients.

MDR UTIs are influenced by unique risk factors in the transplant population and mirror global trends in antimicrobial resistance. While prevalence rates differ across geographic regions, substantial evidence indicates an increasing burden of MDR uropathogens, which now constitute a significant proportion of post-transplant infections [10]. Infections caused by MDR Gram-negative organisms have been linked to increased risks of graft loss, rejection, and systemic complications [11].

Multiple risk factors contribute to MDR UTIs in this population. Prior antibiotic exposure is a major factor, as it facilitates the emergence of resistant organisms [12]. Other healthcare-associated risks include prolonged hospitalization, intensive care unit admission, and urinary catheterization [13]. Transplant-related factors, including delayed graft function, episodes of acute rejection necessitating intensified immunosuppression, and a history of recurrent UTIs, are significant determinants. Additional factors include deceased-donor status, re-transplantation, and the use of immunosuppressive agents such as mycophenolate mofetil (MMF) or antithymocyte globulin (ATG) [1]. Patient-specific factors, such as female sex, diabetes, and advanced age, are also associated with increased susceptibility to MDR UTIs [14].

To date, no published Saudi Arabian study has reported VITEK 2/CLSI-based, species-stratified antimicrobial susceptibility profiles alongside Firth-penalized multivariable predictors of MDR status among first-year KTX recipients. Furthermore, the pronounced and clinically significant divergence in nitrofurantoin activity between *E. coli* and *K. pneumoniae* observed in this study has not been previously documented. The most recent Saudi surveillance data available—a December 2025 study from Al-Baha—included kidney transplant recipients only as a small subset (approximately seven patients), grouped with end-stage renal disease and glomerulonephritis patients into a single ‘renal comorbidity’ subgroup, without transplant-specific antibiogram or predictor analysis [15]. The present study addresses this gap by providing an institution-level antibiogram for KFSHRC, Riyadh, which can be benchmarked against data from Jeddah/Madinah [7], the general population [9,15], and the international literature [5,6]. Additionally, the study identifies UTI recurrence, rather than demographic or transplant-related variables, as the principal correlate of MDR status in this cohort.

## 2. Results

Eligible participants were identified from the records of adult first-time kidney transplant recipients who developed a culture-confirmed Gram-negative UTI within the first year following transplantation at KFSHRC between October 2024 and December 2025. Pediatric recipients, individuals who received a second or subsequent transplant, and episodes involving Gram-positive, fungal, or polymicrobial cultures were excluded. This selection process resulted in a final study sample of 83 recipients, each contributing a single representative Gram-negative UTI episode. The identified uropathogens were *K. pneumoniae* (41 cases; 49.4%), *E. coli* (37 cases; 44.6%), and *P. aeruginosa* (5 cases; 6.0%). MDR organisms accounted for 63.9% of infection episodes (53 of 83; 95% CI 53.1–73.4). The prevalence of MDR organisms was highest in *K. pneumoniae* (29 of 41; 70.7%; 95% CI 55.5–82.4) and *E. coli* (23 of 37; 62.2%; 95% CI 46.1–75.9), with overlapping confidence intervals, and lowest in *P. aeruginosa* (1 of 5; 20.0%). Among MDR isolates, *K. pneumoniae* and *E. coli* comprised 54.7% (29 of 53) and 43.4% (23 of 53), respectively (Figure S1).

### 2.1. Antimicrobial Susceptibility Profiles

Of the 37 *E. coli* isolates, 23 (62.2%) exhibited MDR; nonetheless, susceptibility remained high for several key agents—piperacillin-tazobactam (80.6%), amikacin (94.6%), ertapenem (95.7%), and both imipenem and meropenem (100%)—and, notably, for nitrofurantoin (89.2%), an oral agent not typically regarded as first-line. The highest resistance rates were observed for trimethoprim-sulfamethoxazole (67.6%), cefazolin (67.6%), ciprofloxacin (61.1%), cefotaxime (57.1%), ceftazidime (56.5%), and ceftriaxone (54.1%) (Table 1).

Among 41 *K. pneumoniae* isolates, 29 (70.7%) were classified as MDR; susceptibility to carbapenems and amikacin remained essentially complete (100%), in marked contrast to nitrofurantoin, to which only 15.0% of isolates were susceptible.

The highest resistance rates were observed for trimethoprim-sulfamethoxazole (68.3%), cefotaxime (66.7%), cefazolin (61.0%), ceftazidime (50.0%), ceftriaxone (48.6%), and ciprofloxacin (46.3%) (Table 2).

Of the five *P. aeruginosa* isolates, one (20%) exhibited MDR. *P. aeruginosa* remained susceptible to piperacillin-tazobactam and ceftazidime; however, resistance to ciprofloxacin was 60%, and to carbapenems (imipenem and meropenem) was 33.3% (Table 3).

**Figure 1:**
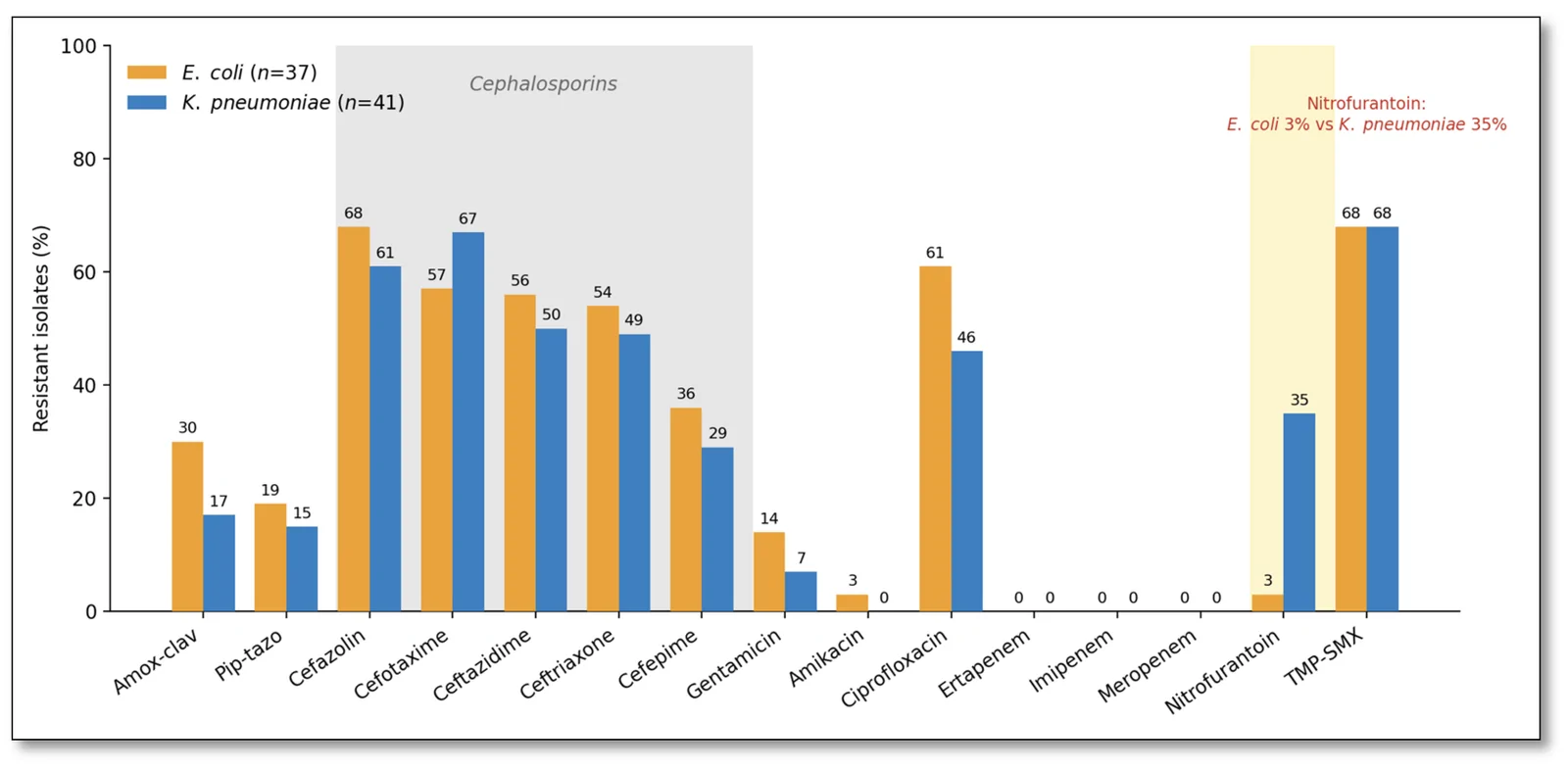
Antimicrobial resistance profiles of *Escherichia coli* (*n* = 37) and *Klebsiella pneumoniae* (*n* = 41). Bars show the percentage of resistant isolates per agent; values are data labels. Carbapenems retained full activity in both species; nitrofurantoin activity diverged sharply (shaded), with resistance of 2.7% in *E. coli* versus 35.0% in *K. pneumoniae*.

Notably, nitrofurantoin activity differed significantly between the two Enterobacterales species: 89.2% of *E. coli* isolates were susceptible compared to 15.0% of *K. pneumoniae* isolates (Fisher *p* < 0.001) (Figure 1).

### 2.2. Comparison of Recipient Characteristics by MDR Status

Recipient and transplant characteristics, including age, sex, donor type, cause of ESRD, immunosuppression, and prophylaxis, were similar between the MDR and non-MDR groups (Table 4). However, UTI recurrence (38/51 [74.5%] vs. 12/30 [40.0%]; *p* = 0.004) and length of hospitalization (median 3.5 vs. 0 days; *p* = 0.045) differed significantly between groups. Symptomatic presentation was less frequent in MDR episodes (*p* = 0.039), consistent with the high proportion of MDR organisms identified in asymptomatic bacteriuria episodes (11/13; 84.6%). Microbiological eradication was achieved less frequently in MDR episodes compared to non-MDR episodes (37/53 [69.8%] vs. 26/30 [86.7%]). Allograft loss occurred in one recipient with MDR-UTI, while no UTI-associated mortality was observed within one year.

### 2.3. Risk Factors Associated with MDR-UTI

**Figure 2:**
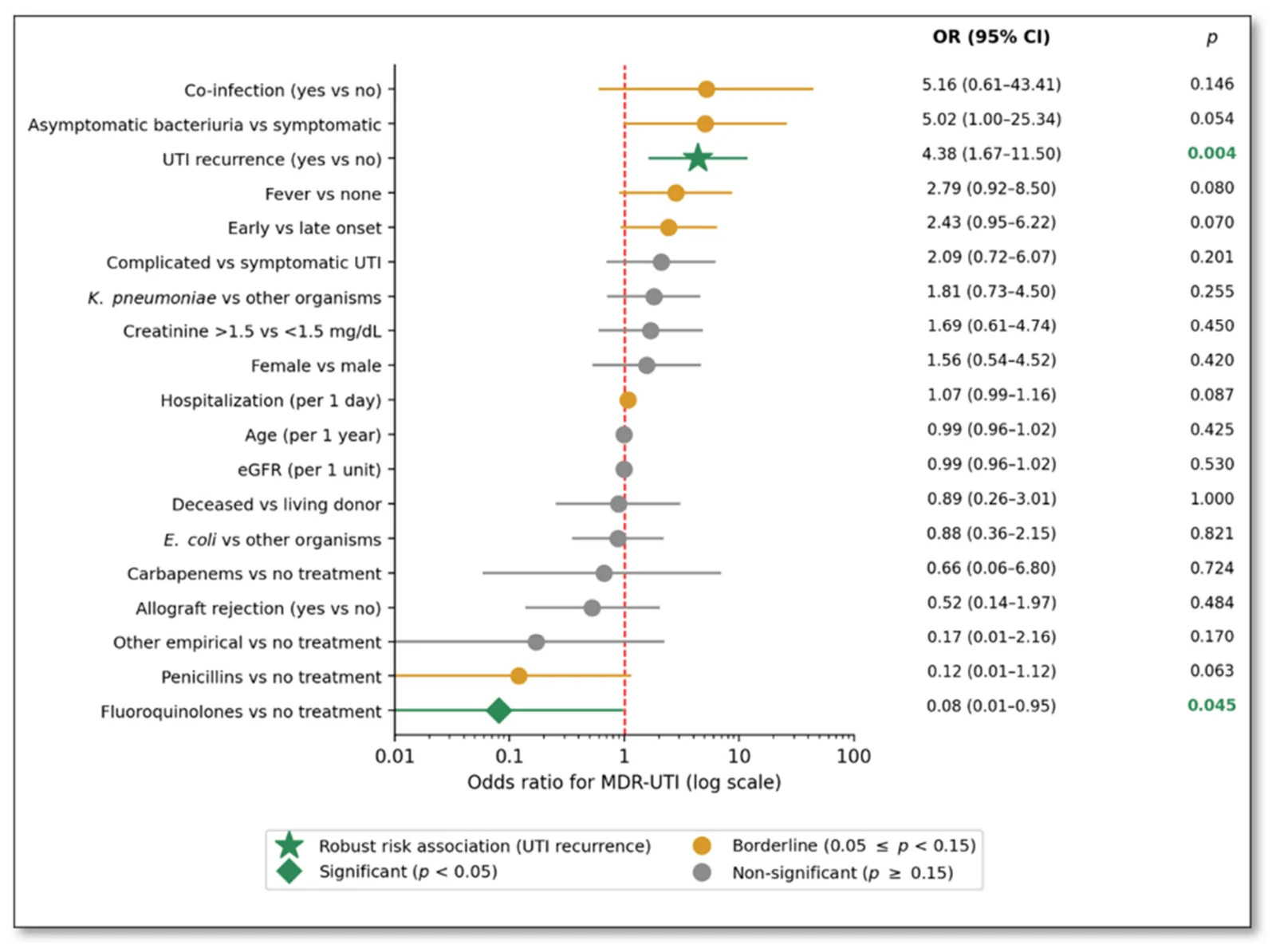
Univariate odds ratios (OR) with 95% confidence intervals for factors associated with MDR-UTI, plotted on a logarithmic scale with the reference line at OR = 1. Estimates are from complete-case analysis. Green denotes statistically significant associations (*p* < 0.05), amber borderline associations (0.05 ≤ *p* < 0.15), and grey non-significant results; the star marks UTI recurrence, the only factor that remained an independent predictor after penalized multivariable adjustment. The inverse association for empirical fluoroquinolone therapy reflects confounding by indication rather than a protective effect.

UTI recurrence was the only factor consistently associated with MDR status. In univariate analysis, UTI recurrence demonstrated a robust association with MDR status (OR 4.38; 95% CI 1.67–11.50; *p* = 0.004), while early-onset infection showed a borderline association (OR 2.43; 95% CI 0.95–6.22; *p* = 0.070) (Table 5, Figure 2). Empirical fluoroquinolone therapy exhibited an inverse association with MDR status (OR 0.083), likely reflecting confounding by indication. Catheter use could not be evaluated because the cohort included no documented non-catheterized recipients. In the penalized multivariable model, UTI recurrence remained the only independent correlate of MDR (aOR 3.99; 95% CI 1.46–10.89; *p* = 0.007), whereas female sex (aOR 2.26; *p* = 0.173) and early-onset infection (aOR 1.95; *p* = 0.192) were not significant (Table 6).

## 3. Discussion

Among kidney transplant recipients in this cohort, nearly two-thirds of first-year Gram-negative urinary isolates exhibited multidrug resistance, indicating a substantial local burden of antimicrobial resistance. Similar rates have been reported in other transplant populations and are generally attributed to recurrent hospitalization, prolonged antibiotic exposure, urinary catheterization, and intensive immunosuppression [16]. This resistance pattern has important therapeutic implications. As resistance to cephalosporins, ciprofloxacin, and trimethoprim-sulfamethoxazole exceeded 45–68%, these agents cannot be considered reliable for empirical therapy. In contrast, the preserved activity of carbapenems and aminoglycosides supports their continued use as dependable, though stewardship-dependent, treatment options.

These findings sit within the heterogeneous regional literature. The overall MDR proportion observed here (63.9%) is considerably higher than the 27.1% reported in a South Asian transplant cohort [8], but broadly consistent with the high resistance burden described in Middle Eastern meta-analytic data [5] and in a recent Saudi multicenter cohort from Jeddah and Madinah. In both the present and the referenced Saudi cohorts, *K. pneumoniae* was the leading uropathogen in contrast to *E. coli*, which is most frequently reported as dominant in international series [5,6,7]. The predominance of *K. pneumoniae* appears to be specific to the transplant population, rather than indicative of broader UTI epidemiology in Saudi Arabia. For example, a large general-population surveillance study from southwestern Saudi Arabia, primarily involving non-transplant patients, identified *E. coli* as the leading uropathogen (47.97%) with a markedly lower MDR burden (30.1% ESBL, 1.9% carbapenemase-producing Enterobacterales) [9]. The recurring predominance of *K. pneumoniae* and higher MDR rates across multiple Saudi tertiary transplant centers, compared with general population data, may reflect the referral-center case mix of Saudi transplant programs, which include higher proportions of complicated, catheter- or stent-associated, and previously antibiotic-exposed episodes, as well as immunosuppressed patients, rather than a generalizable shift in uropathogen ecology. This pattern warrants confirmation in a prospective, multicenter Saudi cohort.

In addition to local surveillance implications, the consistent activity of carbapenems and amikacin across these diverse cohorts [5,6,7,8] supports continued regional reliance on these agents. In contrast, the species-specific divergence in nitrofurantoin susceptibility identified here—not systematically reported in the comparator studies [5,6,7,8]—may offer a carbapenem-sparing oral option for susceptible *E. coli* lower UTI, although other centers should verify this against their own antibiograms.

Among *E. coli* isolates, the relatively low piperacillin-tazobactam resistance suggests that β-lactam/β-lactamase inhibitor combinations may remain effective against certain MDR uropathogens [17,18]. However, the increasing resistance to penicillin derivatives, primarily due to repeated antibiotic exposure and the emergence of ESBLs, remains a significant challenge [19]. The high level of cephalosporin resistance observed, which aligns with findings from previous transplant series [17], likely reflects the widespread presence of ESBL-producing strains [19]. Resistance to fluoroquinolones was also considerable, with 61.1% of isolates resistant to ciprofloxacin, consistent with previous reports linking quinolone resistance to frequent prophylactic and empirical use in transplant populations [18,20]. In contrast, carbapenems (100% susceptibility to imipenem and meropenem) and amikacin (94.6% susceptibility) exhibited strong antimicrobial activity, supporting their continued use as carbapenem- and aminoglycoside-based therapies for resistant Enterobacterales infections [21,22]. However, the potential nephrotoxicity of aminoglycosides requires careful monitoring in this patient group. The high prevalence of resistance to trimethoprim-sulfamethoxazole (67.6%) is clinically important, especially considering its extensive use for prophylaxis [17,18].

Multidrug resistance was observed at an even higher rate among *K. pneumoniae* (70.7%), with increased resistance to cephalosporins, ciprofloxacin, and trimethoprim-sulfamethoxazole, suggesting probable ESBL production [16,17,23]. In contrast, carbapenems and aminoglycosides remained fully effective, consistent with previous studies reporting sustained susceptibility of MDR *K. pneumoniae* to these agents in transplant populations [18,22]. The most clinically significant finding was the marked difference in nitrofurantoin susceptibility between species: 89.2% of *E. coli* isolates were susceptible, compared to only 15.0% of *K. pneumoniae* isolates. Consequently, nitrofurantoin remains an appropriate oral agent for uncomplicated lower urinary tract infections caused by *E. coli,* including multidrug-resistant strains, but should not be used empirically when *K. pneumoniae* is a potential pathogen. Species-specific therapy, informed by culture results and the local antibiogram, is recommended to help preserve carbapenems for the treatment of more severe infections [24]. This species-specific divergence aligns with established differences in nitrofurantoin resistance determinants between these organisms. Nitrofurantoin requires bacterial nitroreductases (NfsA/NfsB) for activation to its bactericidal form, and loss-of-function mutations in these enzymes represent a shared resistance mechanism in both species. However, *K. pneumoniae* possesses additional efflux systems, including AcrAB-TolC and OqxAB, which directly elevate nitrofurantoin minimum inhibitory concentrations (MICs) and are more consistently associated with intrinsic, chromosomally encoded expression in *K. pneumoniae* than in *E. coli*, where similar elevations typically require plasmid-acquired oqxAB [25,26]. Reduced outer-membrane permeability due to porin loss (e.g., OmpK35/OmpK36) has also been described as contributing to the broader resistance phenotype of *K. pneumoniae* relative to *E. coli* [27]. Collectively, these mechanisms provide a molecular explanation for the greater propensity of *K. pneumoniae* isolates to develop nitrofurantoin resistance compared to *E. coli* in this cohort and in similar studies, despite both species sharing the same drug-activation pathway.

Although only 15.0% of *Klebsiella pneumoniae* isolates were susceptible to nitrofurantoin, 50.0% were classified as intermediate according to CLSI criteria. Due to the high urinary concentrations achieved by nitrofurantoin, intermediate isolates may remain clinically treatable for uncomplicated lower urinary tract infections but not for complicated infections. As CLSI M100 does not define a susceptible-dose-dependent (SDD) breakpoint for nitrofurantoin against Enterobacterales, these intermediate classifications reflect standard CLSI interpretive criteria and should be considered when evaluating its empirical therapeutic utility.

Despite the limited sample size of five isolates, *P. aeruginosa* demonstrated significant resistance to ciprofloxacin and carbapenems, whereas piperacillin-tazobactam, ceftazidime, and amikacin remained effective [28]. This observation aligns with previous reports associating increased resistance with prolonged hospitalization and repeated antibiotic exposure [29]. Reporting susceptibility patterns for these infrequent yet clinically significant pathogens contributes to local surveillance and antimicrobial stewardship initiatives [30].

Among clinical correlates, recurrence of UTI was the only factor independently associated with multidrug resistance after penalized adjustment (aOR 3.99). This finding is biologically plausible, as recurrent infection reflects cumulative antimicrobial exposure and repeated healthcare contact [16]. However, the cross-sectional study design precludes causal inference, and recurrence may serve as both a marker and a consequence of resistant infection. Demographic and transplant-related variables highlighted in previous reports, including female sex, were not independent predictors in this dataset. The previously reported sex effect was unstable, likely due to the small number of male recipients (*n* = 18) and residual confounding by clinical presentation and catheter exposure [17]. Catheter use was nearly universal in the available records, preventing evaluation due to the lack of a non-catheterized comparator group. Any observed catheter effect should therefore be considered hypothesis-generating for future research. Similarly, the apparent protective association with empirical fluoroquinolones is most likely attributable to confounding by indication rather than a true microbiological benefit, given that measured fluoroquinolone resistance rates exceeded 45% in Enterobacterales [20].

Episodes of asymptomatic bacteriuria were predominantly multidrug-resistant (84.6%), representing the highest rate among all clinical presentations. Randomized evidence demonstrates that treatment of asymptomatic bacteriuria in KTX recipients does not reduce the incidence of symptomatic infection or rejection; therefore, these colonizing organisms should generally not be treated [31]. However, the potential consequences of untreated MDR colonization, such as subsequent symptomatic infection and transmission of resistance, highlight the need for prospective studies in this immunosuppressed population. MDR episodes were also associated with lower rates of microbiological eradication (69.8% compared to 86.7%) and prolonged hospitalization, underscoring their clinical and economic impact even in the absence of attributable mortality. Notably, the single allograft loss occurred in an MDR recipient, although rejection rates were similar between groups [16,29].

Several limitations should be acknowledged. The study was conducted at a single center with 83 patients and 30 MDR-discordant events, which limited the statistical power to detect only strong associations and precluded stable estimation of weak or separated predictors. Eligible cases were identified directly rather than through screening the entire cohort of transplants performed during the study period. As a result, the number of transplant recipients who did not develop a UTI during the same interval could not be determined, and the total source population was not enumerated. Consequently, estimation of the incidence or prevalence of Gram-negative urinary tract infection among transplant recipients is not possible from these data, and the potential for selection bias remains. Additionally, because case identification focused on culture-confirmed Gram-negative UTI episodes rather than a systematic screen of all urine cultures, the numbers of episodes excluded for Gram-positive, fungal, or polymicrobial growth could not be reliably reconstructed and are not reported. A prospective, fully enumerated screening design would be required to provide these denominators. A recent Saudi cohort study of patients with urinary tract infections and renal comorbidities, including end-stage renal disease, glomerulonephritis, and kidney transplantation, reported that Gram-positive organisms and Candida species together accounted for approximately half of all isolates [15]. This finding suggests that a similar proportion of Gram-negative-excluded episodes may have been present in the source population, although this was not quantified. Catheter use could not be analyzed, and resistance mechanisms were not characterized at the molecular level; therefore, the status for ESBLs and carbapenemases is inferred phenotypically. The retrospective design and per-drug variation in susceptibility testing necessitate cautious, hypothesis-generating interpretation of the risk-factor analysis. A concurrent, hospital-wide (non-transplant) urinary antibiogram was not available for direct comparison in this dataset. Pairing transplant-specific and general hospital antibiograms is recommended to determine whether the resistance patterns identified are unique to the transplant population or indicative of broader institutional or community resistance trends. Such comparative analysis is advised for future institutional surveillance. UTI onset was recorded solely as a binary variable: early (≤6 months) or late (>6 months). More detailed month-by-month stratification, such as first-month versus first-trimester incidence, as reported in some international series, was not available in the source records. Future prospective surveillance should prioritize collecting this level of detail. Structural and functional urological comorbidities recognized as risk factors for urinary tract infection, such as neurogenic bladder, polycystic kidney disease as the native diagnosis, post-surgical urinary incontinence, ureteral stricture, post-void residual urine, and vesicoureteric reflux, were not systematically documented in the source records. Consequently, these variables could not be included as covariates. Their absence should be considered when interpreting the regression estimates in Table 5 and Table 6. Notable strengths include standardized VITEK 2/CLSI testing, complete and internally verified antibiograms, and an analytical approach (penalized regression) suitable for the sample size. Larger, multicenter prospective studies with molecular characterization of resistance and sufficient non-catheterized comparators are required to validate these findings.

## 4. Materials and Methods

### 4.1. Study Design and Population

**Figure 3:**
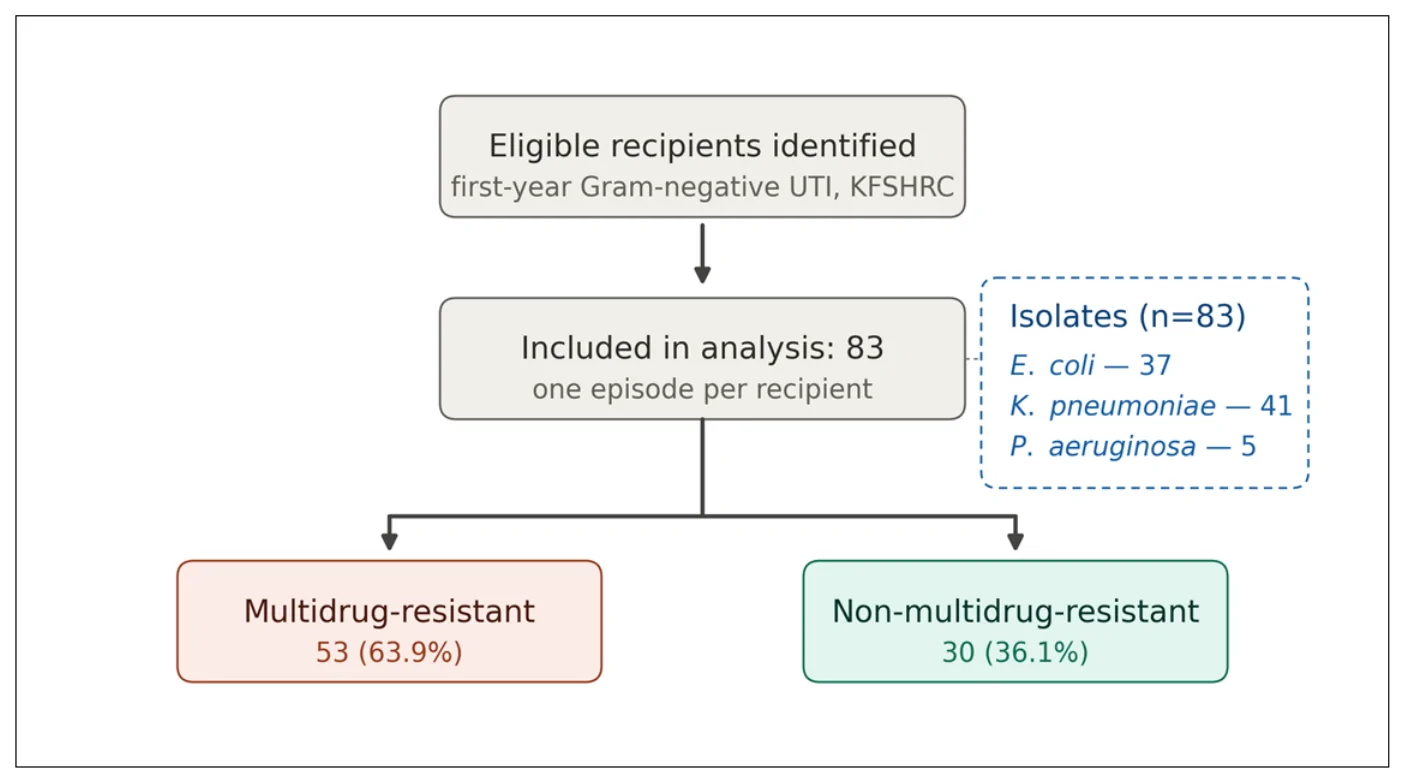
Participant selection and grouping. Episodes involving Gram-positive, fungal, or polymicrobial growth are not reported because these cases were excluded during initial case identification for culture-confirmed Gram-negative monomicrobial urinary tract infection (UTI), rather than being identified through systematic screening of all urine cultures; see Limitations.

This single-center, retrospective, cross-sectional study examined KTX recipients with culture-confirmed Gram-negative UTIs at KFSHRC, Riyadh, Saudi Arabia, between October 2024 and December 2025. The cohort comprised 83 eligible first-time adult KTX recipients who developed a culture-confirmed Gram-negative UTI within the first year post-transplant; each UTI episode was analyzed once per patient. Recipients were identified based on culture-confirmed Gram-negative UTI episodes, rather than through systematic screening of all first-year post-transplant urine cultures. Therefore, the number of Gram-positive, fungal, or polymicrobial episodes encountered during identification was not recorded and cannot be reliably reported. Demographic, transplant-related, and microbiological data were extracted from clinical report files. Recipients were classified asMDR or non-MDR according to the isolate’s susceptibility profile at the time of the episode (Figure 3). Associations with recipient or clinical characteristics should be interpreted cross-sectionally, not as temporal or causal relationships. Ureteral double-J stenting was routinely implemented for nearly all recipients at this center as part of the standard surgical protocol. Stent-removal dates were recorded for 82 of 83 recipients (98.8%), and all but five recipients (94.0%) had documented use of an indwelling urinary catheter. Consequently, a comparator group without catheterization or stenting was not available for regression analysis (see Limitations).

### 4.2. Antimicrobial Susceptibility Testing

Bacterial identification and antimicrobial susceptibility testing (AST) were conducted using the VITEK 2 system (bioMérieux, Marcy-l’étoile, France). Results were interpreted as susceptible, intermediate, or resistant according to the Clinical and Laboratory Standards Institute (CLSI) guidelines (M100, 34th edition (2024) and 35th edition (2025)) [32,33]. The antimicrobial agents tested included penicillins, cephalosporins, aminoglycosides, ciprofloxacin, carbapenems, nitrofurantoin, and trimethoprim-sulfamethoxazole.

Isolates were classified as fully susceptible, non-MDR, or MDR. Susceptible–dose-dependent results were grouped with susceptible for analysis. MDR was defined as non-susceptibility to at least one agent in three or more antimicrobial classes, according to Magiorakos et al. [34] and the European Center for Disease Prevention and Control/Centers for Disease Control and Prevention. Non-MDR isolates were non-susceptible to one or two antibiotic classes. For analyses comparing MDR and non-MDR status, the non-MDR group included both fully susceptible and non-MDR isolates.

### 4.3. Clinical Data Collection

For each recipient, demographics (age and sex) and transplant characteristics (donor type, date of transplantation, cause of ESRD, ATG induction, and maintenance immunosuppression) were recorded. Prophylactic regimens included surgical antibacterial prophylaxis, predominantly cefazolin administered for 3 to 5 days; PJP- prophylaxis with trimethoprim-sulfamethoxazole for 6 to 12 months; CMV- prophylaxis with valganciclovir; and, for deceased-donor recipients, isoniazid prophylaxis. No routine antifungal prophylaxis protocol was implemented in this cohort. Data on urinary catheter use and duration, as well as clinical and laboratory parameters (serum creatinine at the UTI episode, UTI type and post-transplant onset [early, ≤6 months; late, >6 months], co-infection, comorbidities, clinical presentation, empirical and definitive antibiotic regimens and duration, and microbiological eradication) were collected. Outcomes assessed included UTI recurrence, length of hospitalization, allograft rejection and loss, eGFR at one year, and one-year UTI-associated mortality.

### 4.4. Statistical Analysis

Analyses were conducted using R version 4.3.2 (R Foundation for Statistical Computing, Vienna, Austria). Continuous variables were summarized as mean ± standard deviation (SD) or median (interquartile range, IQR), depending on their distribution, and compared between MDR and non-MDR groups using the independent-samples t-test or the Mann–Whitney U test, respectively. Categorical variables were summarized as frequencies and percentages with 95% confidence intervals (CIs) and compared using the Pearson χ^2^ test, or Fisher’s exact test when any expected cell count was below five. Missing data were addressed through complete-case analysis without imputation; the number of recipients with available data is reported for each variable, and all percentages were calculated using the variable-specific denominator. Factors associated with MDR-UTI were initially examined by univariable logistic regression. Variables that were clinically relevant or associated with MDR status at the univariable level were subsequently included in a multivariable model fitted by Firth’s penalized-likelihood logistic regression to reduce small-sample bias and address separation. Catheter use was documented for descriptive analysis only and was excluded from comparative and regression analyses. Because all recipients with available data had catheters, the lack of a non-catheterized comparator prevented estimation of its association with MDR status (quasi-complete separation). Odds ratios (OR) and adjusted odds ratios (aOR) are reported with 95% CIs. All tests were two-sided, and *p* < 0.05 was considered statistically significant.

## 5. Conclusions

Among kidney-transplant recipients, Gram-negative UTIs during the first year were predominantly caused by MDR *K. pneumoniae* and *E. coli*, with MDR identified in nearly two-thirds of episodes. High resistance rates to cephalosporins, fluoroquinolones, and trimethoprim-sulfamethoxazole rendered these agents unreliable for empirical therapy. In contrast, carbapenems and aminoglycosides maintained strong activity and should be preserved through active stewardship. The pronounced species-specific difference in nitrofurantoin activity emphasizes the importance of culture-guided, species-directed treatment. After adjusting for data limitations and applying penalized regression, UTI recurrence, rather than demographic or device-related factors, was identified as the principal correlate of multidrug resistance. The elevated MDR rate in asymptomatic bacteriuria, along with lower eradication rates and prolonged hospitalization observed in MDR episodes, demonstrates the clinical burden of resistance even in the absence of attributable mortality. Ongoing local surveillance, early risk stratification, minimization of unnecessary catheterization, and implementation of institution-specific antimicrobial stewardship programs are essential to improve post-transplant UTI management and preserve graft function. Larger multicentre prospective studies incorporating molecular characterization of resistance mechanisms are necessary to validate these findings.

## Figures and Tables

**Table 1:** Pattern of antimicrobial resistance in 37 *E. coli* clinical isolates used in the study.

Antimicrobial Category	Antimicrobial Agent	Non MDR *n* (%)	MDR *n* (%)	
Multidrug Resistance		14 (37.8%)	23 (62.2%)	
		Susceptible *n* (%)	Intermediate *n* (%)	Resistant *n* (%)
Penicillins	Amoxicillin and Clavulanate	23 (62.2%)	3 (8.1%)	11 (29.7%)
	Piperacillin-tazobactam (*n* = 36)	29 (80.6%)	0 (0.0%)	7 (19.4%)
Cephalosporins	Cefazolin	12 (32.4%)	0 (0.0%)	25 (67.6%)
	Cefotaxime (*n* = 14)	6 (42.9%)	0 (0.0%)	8 (57.1%)
	Ceftazidime (*n* = 23)	5 (21.7%)	5 (21.7%)	13 (56.5%)
	Ceftriaxone	17 (45.9%)	0 (0.0%)	20 (54.1%)
	Cefepime (*n* = 36)	23 (63.9%)	0 (0.0%)	13 (36.1%)
Aminoglycosides	Amikacin	35 (94.6%)	1 (2.7%)	1 (2.7%)
	Gentamicin	32 (86.5%)	0 (0.0%)	5 (13.5%)
Quinolones	Ciprofloxacin (*n* = 36)	5 (13.9%)	9 (25%)	22 (61.1%)
Carbapenems	Ertapenem (*n* = 23)	22 (95.7%)	1 (4.3%)	0 (0.0%)
	Imipenem (*n* = 24)	24 (100%)	0 (0.0%)	0 (0.0%)
	Meropenem (*n* = 25)	25 (100%)	0 (0.0%)	0 (0.0%)
Nitrofuran	Nitrofurantoin	33 (89.2%)	3 (8.1%)	1 (2.7%)
Sulphonamides	Trimethoprim-sulfamethoxazole	12 (32.4%)	0 (0.0%)	25 (67.6%)

**Table 2:** Pattern of antimicrobial resistance in 41 *K. pneumoniae* clinical isolates used in the study.

Antimicrobial Category	Antimicrobial Agent	Non MDR *n* (%)	MDR *n* (%)	
Multidrug Resistance		12 (29.3%)	29 (70.7%)	
		Susceptible *n* (%)	Intermediate *n* (%)	Resistant *n* (%)
Penicillins	Amoxicillin and Clavulanate	31 (75.6%)	3 (7.3%)	7 (17.1%)
	Piperacillin-tazobactam	33 (80.5%)	2 (4.9%)	6 (14.6%)
Cephalosporins	Cefazolin	16 (39%)	0 (0.0%)	25 (61%)
	Cefotaxime (*n* = 15)	4 (26.7%)	1 (6.7%)	10 (66.7%)
	Ceftazidime (*n* = 20)	2 (10%)	8 (40%)	10 (50%)
	Ceftriaxone (*n* = 37)	19 (51.4%)	0 (0.0%)	18 (48.6%)
	Cefepime	29 (70.7%)	0 (0.0%)	12 (29.3%)
Aminoglycosides	Amikacin (*n* = 40)	40 (100%)	0 (0.0%)	(0.0%)
	Gentamicin	38 (92.7%)	0 (0.0%)	3 (7.3%)
Quinolones	Ciprofloxacin	12 (29.3%)	10 (24.4%)	19 (46.3%)
Carbapenems	Ertapenem (*n* = 20)	20 (100%)	0 (0.0%)	0 (0.0%)
	Imipenem (*n* = 23)	23 (100%)	0 (0.0%)	0 (0.0%)
	Meropenem (*n* = 23)	23 (100%)	0 (0.0%)	0 (0.0%)
Nitrofuran	Nitrofurantoin (*n* = 40)	6 (15%)	20 (50%)	14 (35%)
Sulphonamides	Trimethoprim-sulfamethoxazole	13 (31.7%)	0 (0.0%)	28 (68.3%)

**Table 3:** Pattern of antimicrobial resistance in 5 *P. aeruginosa* clinical isolates used in the study.

Antimicrobial Category	Antimicrobial Agent	Non MDR *n* (%)	MDR *n* (%)	
Multidrug Resistance		4 (80%)	1 (20%)	
		Susceptible *n* (%)	Intermediate *n* (%)	Resistant *n* (%)
Penicillins	Amoxicillin and Clavulanate (*n* = 1)	0 (0.0%)	0 (0.0%)	1 (100%)
	Piperacillin-tazobactam (*n* = 4)	4 (100%)	0 (0.0%)	0 (0.0%)
Cephalosporins	Cefazolin (*n* = 1)	0 (0.0%)	0 (0.0%)	1 (100%)
	Ceftazidime (*n* = 4)	4 (100%)	0 (0.0%)	0 (0.0%)
	Cefepime	4 (80%)	1 (20.0%)	0 (0.0%)
Aminoglycosides	Amikacin	4 (80%)	1 (20.0%)	0 (0.0%)
Quinolones	Ciprofloxacin	2 (40%)	0 (0.0%)	3 (60%)
Carbapenems	Imipenem (*n* = 3)	0 (0.0%)	2 (66.7%)	1 (33.3%)
	Meropenem (*n* = 3)	2 (66.7%)	0 (0.0%)	1 (33.3%)

**Table 4:** Demographic and clinical characteristics of kidney transplant recipients and associated uropathogens, categorized by multidrug-resistant (MDR) status.

Characteristic	Total (*n* = 83)	Non-MDR (*n* = 30)	MDR (*n* = 53)	*p* Value
	***n*** **(%)**	***n*** **(%)**	***n*** **(%)**	
**Age, years—median (IQR)**	52 (36–61)	53 (38–61)	50 (35–61)	0.385
**Sex**				**0.420**
Female	65 (78.3)	22 (73.3)	43 (81.1)	
Male	18 (21.7)	8 (26.7)	10 (18.9)	
**Donor type**				**1.000**
Living	70 (84.3)	25 (83.3)	45 (84.9)	
Deceased	13 (15.7)	5 (16.7)	8 (15.1)	
**ESRD cause**				**0.923**
Glomerulonephritis	22 (26.5)	7 (23.3)	15 (28.3)	
Diabetes mellitus	19 (22.9)	8 (26.7)	11 (20.8)	
Hypertension	13 (15.7)	4 (13.3)	9 (17.0)	
Obstructive	9 (10.8)	4 (13.3)	5 (9.4)	
Other/unknown	20 (24.1)	7 (23.3)	13 (24.5)	
**ATG induction**	72 (86.7)	28 (93.3)	44 (83.0)	0.313
**Maintenance immunosuppression**				**0.504**
Tacrolimus/MMF/prednisone	78 (94.0)	28 (93.3)	50 (94.3)	
Tacrolimus/azathioprine/pred.	3 (3.6)	2 (6.7)	1 (1.9)	
Sirolimus/prednisone	1 (1.2)	0 (0)	1 (1.9)	
**Surgical prophylaxis**				**1.000**
Cefazolin	50 (60.2)	18 (60.0)	32 (60.4)	
Piperacillin-tazobactam	12 (14.5)	4 (13.3)	8 (15.1)	
Not documented	21 (25.3)	8 (26.7)	13 (24.5)	
**PJP prophylaxis**	79 (95.2)	29 (96.7)	50 (94.3)	1.000
**CMV prophylaxis**	79 (95.2)	30 (100)	49 (92.5)	0.291
**Isoniazid prophylaxis (deceased donor)**	14 (16.9)	5 (16.7)	9 (17.0)	1.000
**Catheter duration, days—median (IQR)**	6 (5–14)	7 (5–14)	5 (5–11)	0.667
**Creatinine at UTI episode (*n* = 80) ^a^**				**0.450**
<1.5 mg/dL	56 (70.0)	23 (76.7)	33 (66.0)	
>1.5 mg/dL	24 (30.0)	7 (23.3)	17 (34.0)	
**UTI type (*n* = 81) ^b^**				**0.067**
Symptomatic UTI	44 (54.3)	21 (70.0)	23 (45.1)	0.039 **^c^**
Complicated UTI	24 (29.6)	7 (23.3)	17 (33.3)	
Asymptomatic bacteriuria	13 (16.0)	2 (6.7)	11 (21.6)	0.117 **^c^**
**UTI onset (*n* = 81) ^d^**				**0.070**
Early (≤6 months)	38 (46.9)	10 (33.3)	28 (54.9)	
Late (>6 months)	43 (53.1)	20 (66.7)	23 (45.1)	
**Co-infection**	9 (10.8)	1 (3.3)	8 (15.1)	0.146
**Comorbidities**				
Hypertension	49 (59.0)	20 (66.7)	29 (54.7)	0.356
Diabetes mellitus	13 (15.7)	4 (13.3)	9 (17.0)	0.761
Malignancy	2 (2.4)	1 (3.3)	1 (1.9)	1.000
**Clinical presentation**				**0.150**
Fever	24 (28.9)	5 (16.7)	19 (35.8)	0.080 **^c^**
Asymptomatic	54 (65.1)	23 (76.7)	31 (58.5)	
GIT/respiratory	5 (6.0)	2 (6.7)	3 (5.7)	
**Empirical antibiotic (*n* = 81) ^e^**				0.027 *
Carbapenem	25 (30.9)	4 (13.3)	21 (41.2)	
Amoxicillin-clavulanate	32 (39.5)	16 (53.3)	16 (31.4)	
Fluoroquinolone	10 (12.3)	6 (20.0)	4 (7.8)	
Other	7 (8.6)	3 (10.0)	4 (7.8)	
None (untreated)	7 (8.6)	1 (3.3)	6 (11.8)	
**Empirical = definitive regimen ^f^**	68 (81.9)	25 (83.3)	43 (81.1)	1.000
**Duration of antibiotic therapy (*n* = 74) ^g^**				
≤7 days	27 (36.5)	13 (44.8)	14 (31.1)	0.145
10–14 days	44 (59.5)	16 (55.2)	28 (62.2)	1.000
14–21 days	3 (4.1)	0 (0)	3 (6.7)	0.550
**Organism**				**0.080**
*E. coli*	37 (44.6)	14 (46.7)	23 (43.4)	
*K. pneumoniae*	41 (49.4)	12 (40.0)	29 (54.7)	
*P. aeruginosa*	5 (6.0)	4 (13.3)	1 (1.9)	
**Microbiological eradication**	63 (75.9)	26 (86.7)	37 (69.8)	0.111
**UTI recurrence (*n* = 81) ^h^**	50 (61.7)	12 (40.0)	38 (74.5)	0.004 *
**Length of hospitalization, days—median (IQR)**	0 (0–10)	0 (0–4)	3.5 (0–14)	0.045 *
**Allograft rejection**	10 (12.0)	5 (16.7)	5 (9.4)	0.484
**Allograft loss at 1 yr**	1 (1.2)	0 (0)	1 (1.9)	1.000
**eGFR at 1 yr, mL/min/1.73 m^2^—median (IQR)**	61 (46–61)	61 (52–61)	61 (45–61)	0.478
**UTI-associated mortality at 1 yr**	0 (0)	0 (0)	0 (0)	—

**Footnotes.** Data is presented as *n* (column %) or median (IQR). Percentages were calculated using the number of recipients with non-missing data for each variable (complete-case analysis; no values were imputed). As a result, group denominators may differ from the column header where data were missing, and these are indicated in the relevant variable label. Binary variables are reported with both levels under a single variable-level *p*-value. *p* values are derived from Fisher’s exact test for dichotomous variables, the χ^2^ test for polytomous (omnibus) variables, and the Mann–Whitney U test for continuous variables (all right-skewed). **^a^** Creatinine was missing for three recipients (all in the MDR group); group denominators are 50 (MDR) and 30 (non-MDR). **^b^** UTI type was missing for two recipients (both MDR); group denominators are 51 (MDR) and 30 (non-MDR). **^c^** Category-versus-rest comparison (within complete cases). **^d^** UTI onset was missing for two recipients (both MDR); group denominators are 51 (MDR) and 30 (non-MDR). **^e^** Two recipients (both MDR) had no documented empirical regimen and were excluded; if these episodes are confirmed as genuinely untreated rather than undocumented, they may instead be reported within “None (untreated)” (group denominators would then revert to 53/30). **^f^** Exact-match concordance across all episodes; among treated episodes only, concordance was 63 of 74 (85.1%). **^g^** Nine untreated/asymptomatic episodes had no therapy duration. **^h^** UTI recurrence status was missing for two recipients (both MDR); group denominators are 51 (MDR) and 30 (non-MDR). Abbreviations: MDR, multidrug-resistant; ESRD, end-stage renal disease; ATG, antithymocyte globulin; MMF, mycophenolate mofetil; PJP, *Pneumocystis jirovecii* pneumonia; CMV, cytomegalovirus; eGFR, estimated glomerular filtration rate; IQR, interquartile range. * Significant at 0.05 level.

**Table 5:** Results of univariate logistic regression analysis examining factors associated with MDR-UTI.

Variable (Comparison)	OR (95% CI)	*p* Value	*N*
UTI recurrence (yes vs. no)	4.38 (1.67–11.50)	0.004 *	81
Asymptomatic bacteriuria vs. symptomatic	5.02 (1.00–25.34)	0.054	74
Co-infection (yes vs. no)	5.16 (0.61–43.41)	0.146	83
Fever vs. none	2.79 (0.92–8.50)	0.080	83
Early vs. late onset	2.43 (0.95–6.22)	0.070	81
Complicated vs. symptomatic UTI	2.09 (0.72–6.07)	0.201	81
*K. pneumoniae* vs. other organisms	1.81 (0.73–4.50)	0.255	83
Creatinine >1.5 vs. <1.5 mg/dL	1.69 (0.61–4.74)	0.450	80
Female vs. male	1.56 (0.54–4.52)	0.420	83
Hospitalization (per 1 day)	1.073 (0.990–1.164)	0.087	82
Age (per 1 year)	0.989 (0.961–1.017)	0.425	83
eGFR (per 1 unit)	0.989 (0.956–1.023)	0.530	81
Deceased vs. living donor	0.89 (0.26–3.01)	1.000	83
*E. coli* vs. other organisms	0.88 (0.36–2.15)	0.821	83
Allograft rejection (yes vs. no)	0.52 (0.14–1.97)	0.484	83
Empirical antibiotic (ref = no treatment)			
Penicillins	0.125 (0.014–1.118)	0.063	81
Carbapenems	0.656 (0.063–6.797)	0.724	81
Fluoroquinolones	0.083 (0.007–0.950)	0.045 *	81
Other	0.167 (0.013–2.160)	0.170	81

**Footnotes.** OR odds ratio for MDR status; CI, confidence interval. Categorical ORs are derived from 2 × 2 contingency tables (Haldane–Anscombe correction applied only where a zero-cell occurred); continuous ORs are from univariate logistic regression. Estimates use recipients with non-missing data for the respective variable (complete-case analysis). The inverse fluoroquinolone association reflects confounding by indication rather than a protective effect. * Significant at 0.05 level.

**Table 6:** Penalized multivariable logistic regression identifying independent predictors of MDR-UTI.

Predictor	Adjusted OR (95% CI)	*p* Value	Note
UTI recurrence (yes vs. no)	3.99 (1.46–10.89)	0.007 *	Independent predictor
Female vs. male	2.26 (0.70–7.34)	0.173	NS
Early vs. late onset	1.95 (0.72–5.33)	0.192	NS

**Footnotes.** Firth penalized-likelihood logistic regression on complete cases (*n* = 81; two recipients with missing recurrence/onset data excluded); 30 minority-class events, 3 covariates (≈10 events per variable). Unpenalized maximum-likelihood estimates were nearly identical (recurrence aOR 4.30, *p* = 0.005; female 2.37, *p* = 0.155; early-onset 2.02, *p* = 0.174), supporting the stability of the estimates. Recurrence may represent both a marker and a consequence of MDR infection; the temporal relationship cannot be determined within this cross-sectional design. NS, not significant. * Significant at 0.05 level.

## Data Availability

The datasets generated and analyzed during this study are not publicly available due to institutional data protection policy. However, they may be obtained from Fatimah Alhamlan (Department of Infection and Immunity, King Faisal Specialist Hospital and Research Center, Riyadh, Saudi Arabia; falhamlan@kfshrc.edu.sa) upon reasonable request and subject to approval in accordance with KFSHRC data sharing policy.

## Supplementary Materials

The following supporting information can be downloaded at https://www.mdpi.com/article/10.3390/antibiotics15080769/s1, Figure S1: Prevalence of multidrug resistance among uropathogens. Bars show MDR prevalence with Wilson score 95% confidence intervals; numerators/denominators are labelled. The dashed line marks the overall MDR prevalence (63.9%).

## Abbreviations

The following abbreviations are used in this manuscript:

MDR	Multi-drug resistance
UTIs	Urinary tract infections
KTX	Kidney transplant
KFSHRC	King Faisal specialist hospital and research center
MMF	mycophenolate mofetil
ATG	antithymocyte globulin
ESRD	end-stage renal disease
AST	Antimicrobial susceptibility test
aOR	Adjusted odds ratio
CI	Confidence interval
SD	Standard deviation
CMV	Cyto megalo virus
ESBLs	Extended spectrum beta lactamases
PJP	*Pneumocystis jirovecii* pneumonia
CLSI	Clinical Laboratory Standards Institute
MICs	minimum inhibitory concentrations
