# Supplementary material for: Nitrofurantoin Susceptibility and the Landscape of Multidrug-Resistant Gram-Negative Uropathogens in Kidney Transplant Recipients During the First Post-Transplant Year
Source: Antibiotics (Basel). 2026 Aug 10;15(8):769. doi: 10.3390/antibiotics15080769 (PMC13510060; doi:10.3390/antibiotics15080769)
Supplement: Supplementary file 1 [file antibiotics-15-00769-s001.zip › antibiotics-4417769-supplementary.pdf]

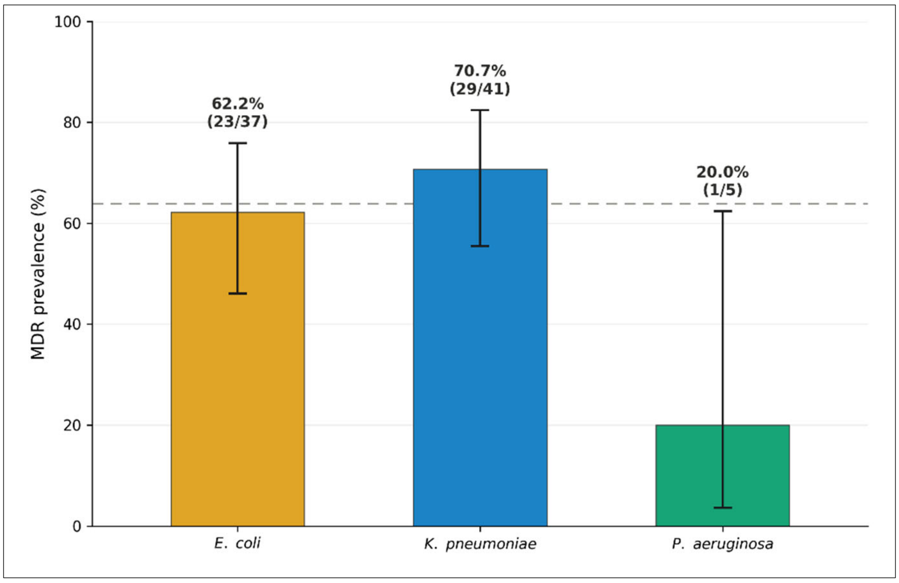

Figure S1. Prevalence of multidrug resistance among uropathogens. Bars show MDR prevalence with Wilson score 95% confidence intervals; numerators/denominators are labelled. The dashed line marks the overall MDR prevalence (63.9%).

Plotting dataset (Wilson 95% CI)

Organism	MDR/total	% MDR	95% CI	CI method
E. coli	23/37	62.2	46.1–75.9	Wilson score
K. pneumoniae	29/41	70.7	55.5–82.4	Wilson score
P. aeruginosa	1/5	20.0	3.6–62.4	Wilson score
**Overall**	53/83	63.9	53.1–73.4	Wilson score
